# Distinct B Cell Receptor Functions Are Determined by Phosphorylation

**DOI:** 10.1371/journal.pbio.0040231

**Published:** 2006-05-30

**Authors:** Richard Robinson

The B cell receptor (BCR) stands sentry on the front lines of the body's defenses against infection. Embedded in the surface of the B cell—one of the principal immune cells—its job is to bind foreign substances called antigens. The binding of an antigen sounds the alarm that a pathogen has invaded and activates the immune system.

As a group, BCRs do two things in response to antigen binding—internalization and signaling. These functions are accomplished by different portions of the receptor: the immunoglobulin chain, which projects out of the cell, and the signaling chains, which face inward. Internalization of BCRs drags their bound antigens with them. Inside the cell, the antigen is processed into fragments that will ultimately be presented again at the surface to stimulate T cells, orchestrators of the full-fledged immune response. BCRs also begin signaling on the cell surface, preparing the B cell for efficient antigen presentation. The relationship between these two processes of internalization and signaling, and whether each receptor does both, has been unclear. In a new study, Ping Hou, Aaron Dinner, Marcus Clark, and colleagues show that the processes are distinct, and that, although most receptors are internalized, a few remain on the surface to initiate signaling. Which path a receptor takes is determined by whether or not it becomes phosphorylated immediately after binding antigen.

Phosphorylation, or the addition of phosphate groups, is a ubiquitous means of protein regulation, often switching it from one function to another. Phosphates are attached to specific amino acids, including tyrosine. The authors began by replacing tyrosine residues on the cytoplasmic tail of the BCR with one of two other amino acids, alanine or phenylalanine. They found that phenylalanine, which is structurally similar to tyrosine but cannot be phosphorylated, still allowed internalization, whereas alanine delayed it. This suggested that the endocytosis, or internalization, machinery might bind to the unphosphorylated tyrosine residue with adaptor molecules to begin the internalization process; the ability to do the same to phenylalanine but not to alanine likely explained the difference in results. The researchers showed that the effect is mediated not only by tyrosine residues in a well-recognized motif within the receptor tail, but also—and even more so—by tyrosines that are independent of this region and whose function had not previously been well characterized.

Further direct support for the link between the phosphorylation state and internalization came from direct visualization with a stain specific for the phosphorylated receptor complex. These complexes were never found inside the cell, but instead were clustered on the surface, along with multiple unphosphorylated receptors. The researchers next asked whether surface or internalized BCRs were involved in signaling. By inhibiting internalization and measuring the effect on activities downstream from the receptor trigger, they showed that internalized BCRs play little role in stimulating these activities.

Finally, they constructed a simple mathematical model of the competition for receptor tyrosines between the kinase enzyme that adds the phosphate and the adaptor molecules that link to the unphosphorylated tyrosines to promote internalization. They showed that the either/or fate of the BCR—remaining on the surface to engage in signaling or becoming internalized—enhances responses to weak antigens, which may be an important feature for initial antigen detection. They also describe how the model accounts for several other formerly puzzling features of the antigen response by B cells.

The results of these experiments can be used to clarify the dynamics of activated BCRs, both on the surface and inside the cell. This should lead to a better understanding both of receptor-mediated signaling and the processing of internalized receptors—both of which are crucial for an effective immune response.

## 

**Figure:**
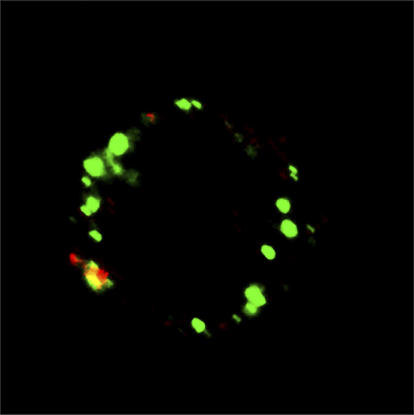
This B cell is stained for immunoglobulin (Ig) (green) and a probe that detects phosphorylated Iga (red). Phosphorylated BCR complexes (yellow) are found at the surface of the cell.

